# The Molecular Structure, Expression, and Emerging Role of the 15-Leucine-Rich Repeat Containing Membrane Protein (LRRC15) in Skeletal Biology and Diseases

**DOI:** 10.3390/ijms27156582

**Published:** 2026-07-24

**Authors:** Zi-Hao Lin, Zhi-Chao Hu, Chang-Qing Zhang, Zhen-Zhong Zhu, Qian Tang

**Affiliations:** Department of Orthopedic Surgery, Shanghai Sixth People’s Hospital Affiliated to Shanghai Jiao Tong University School of Medicine, 600 Yishan Road, Shanghai 200233, Chinazhangcq@sjtu.edu.cn (C.-Q.Z.)

**Keywords:** LRRC15, cell signaling, extracellular matrix, skeletal biology, osteosarcoma

## Abstract

The 15-leucine-rich repeat containing membrane protein (LRRC15) is a transmembrane protein derived from the leucine-rich repeat (LRR) superfamily that participates in various cell–cell and cell–extracellular matrix interactions, regulating fibrogenesis, vascular invasion, tumorigenesis, and the formation of the innate immune barrier to drive specific antiviral responses. LRRC15, whose expression is reportedly induced by TGF-β and IL-1β stimulation, was recently found to be associated with the development of skeletal diseases, and its presence has been related to the pathophysiological processes of osteoporosis, osteoarthritis and rheumatoid arthritis. Various transmembrane and intracellular signaling transductions are involved in LRRC15-mediated molecular functions, including integrin, NF-κB, Wnt/β-catenin, etc. Additionally, LRRC15 is considered a novel mesenchymal protein and an important biomarker for the occurrence and development of osteosarcoma. Understanding the role of LRRC15 in the bone, joint and tumor microenvironment will facilitate the development of new therapeutic options—particularly LRRC15-based target therapies—for skeletal diseases.

## 1. Introduction

Leucine-rich repeats (LRRs) are a superfamily of highly conserved protein–ligand interaction motifs that play fundamental roles in extracellular matrix (ECM) biology [1]. Numerous LRR family members have been implicated in skeletal development and homeostasis, contributing to the regulation of bone and cartilage physiology as well as the pathogenesis of musculoskeletal disorders [2,3,4].

As another important member of the LRR superfamily, the 15-leucine-rich repeat containing membrane protein (LRRC15, also known as lib) is a type I transmembrane protein that mediates cell adhesion and participates in diverse physiological and pathological processes. Originally identified as a β-amyloid-inducible gene in neuroinflammation, LRRC15 has since been implicated in broad functional domains: ECM interactions and cytokine signaling in specialized tissue niches such as hair follicles, tonsils, and wound-healing sites [5,6]; innate immune responses, including inhibition of adenovirus infection and blockade of SARS-CoV-2 entry [7,8]; and tumor biology, where it is highly expressed on cancer-associated fibroblasts (CAFs) and promotes metastasis in ovarian cancer and triple-negative breast cancer (TNBC) through β1-integrin/FAK and Wnt/β-catenin signaling [9,10]. In addition, early evidence has indicated that LRRC15 is highly expressed in the pulpal tissue of caries lesions, suggesting that it may play a role in mineralized tissue-related disease [11]. Notably, LRRC15 expression is extremely restricted in normal tissues, which distinguishes it from ubiquitous adhesion molecules and renders it an attractive therapeutic target [6].

In recent years, accumulating evidence has established LRRC15 as a potent regulator of skeletal biology. Firstly, LRRC15 enhances osteogenic differentiation of mesenchymal stromal cells (MSCs) and attenuates osteoporosis (OP) through suppression of NF-κB signaling [12]. Secondly, LRRC15 is markedly upregulated in osteoarthritic cartilage, where it is induced by inflammatory cytokines and DNA demethylation to drive pathological cartilage catabolism [13]. Thirdly, LRRC15 overexpression in rheumatoid arthritis (RA) synovial fibroblasts promotes joint inflammation through a RUNX1-dependent transcriptional mechanism [14]. Furthermore, LRRC15 is highly expressed in osteosarcoma (OS) and serves as a prognostic biomarker associated with metastatic potential and chemotherapy resistance [6,15,16].

In this review, we describe the molecular structure of LRRC15, investigate its expression, and discuss its role in cell signaling transduction and skeletal biology. Although the underlying molecular mechanism of LRRC15 in skeletal biology has yet to be fully elucidated, LRRC15-targeting drugs hold excellent potential as therapeutic agents, with the capacity to ameliorate LRRC15-mediated skeletal diseases.

## 2. Tissue Expression Patterns and Cellular Localization of LRRC15

### 2.1. Normal Tissue Distribution

LRRC15 displays a highly restricted expression profile in healthy human tissues, a feature that distinguishes it from ubiquitously expressed cell adhesion molecules and establishes it as a definitive marker of specialized cellular states [6]. Data from the latest releases of the Human Protein Atlas (HPA) and Genotype-Tissue Expression (GTEx) Portal confirm that LRRC15 mRNA is present at low to undetectable levels across the vast majority of adult human tissues, with the only notable exceptions being hair follicles, tonsillar tissue, and the placenta [6,17,18]. In the periodontal ligament (PDL), LRRC15 is expressed by resident fibroblasts and is required for maintaining PDL fibroblast adhesion to the ECM and migratory capacity [19].

This restricted expression pattern has important therapeutic implications, as it provides a favorable window for LRRC15-targeted therapies with minimal risk of on-target, off-tissue effects.

### 2.2. Expression in Skeletal System

In the skeletal system, LRRC15 is expressed at negligible levels in mature, homeostatic adult bone. This is consistent with its function as an activation-associated marker rather than as a constitutive structural protein. By contrast, during states of active skeletal remodeling, including fracture repair and endochondral ossification, LRRC15 is robustly and transiently upregulated in periosteal progenitor cells (PPCs) and MSCs [12,20]. This dynamic expression pattern suggests that LRRC15 functions as a context-dependent regulator of skeletal tissue remodeling rather than a housekeeping gene.

In healthy articular cartilage, LRRC15 is virtually undetectable, but its expression is markedly upregulated in osteoarthritic cartilage, most notably in regions undergoing pathological ossification and chondrocyte hypertrophy [13,21]. A comparable expression pattern is observed in synovial tissue: LRRC15 is expressed at low levels in healthy joints, but is strongly induced in the synovium of patients with RA, where it specifically labels activated fibroblast-like synoviocytes (FLSs) [14].

**Figure 1:**
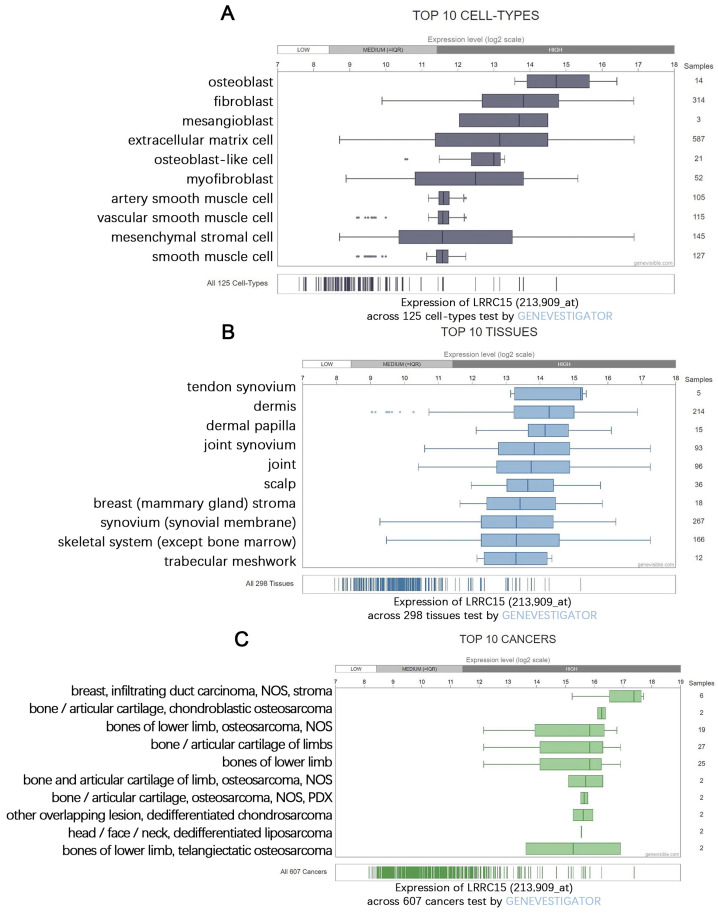
mRNA expression profiling of LRRC15 gene in humans. It ranks among the top 10 most abundantly expressed cell-types (**A**), tissues (**B**) and cancers (**C**), as predicted and assembled by Genevisible^®^ bioinformatics tool (accessed on 6 October 2023).

However, expression profiling analyses consistently demonstrate a strong correlation between LRRC15 and the skeletal system. According to Genevisible^®^ gene profiling (accessed on 6 October 2023), LRRC15 mRNA ranks among the most abundantly expressed transcripts in musculoskeletal tissues and cells, including osteoblasts, myofibroblasts, mesenchymal stromal cells, tendon synovium, and joint synovium (Figure 1A,B) [6]. Meanwhile, LRRC15 is reported to be frequently overexpressed in various tumor-related cells, such as on stromal fibroblasts in many solid tumors and directly on mesenchymal-derived cancer cells [5,6,16]. However, according to the predictive analysis, with the exception of breast stroma cancers, LRRC15 is highly prevalent in bone- or cartilage-related tumors (Figure 1C). These results indicate that there is a strong correlation between LRRC15 and skeletal diseases.

### 2.3. Single-Cell Transcriptomic Profiling

Single-cell RNA sequencing (scRNA-seq) has transformed our understanding of LRRC15 expression at a single-cell resolution, definitively demonstrating that LRRC15 specifically identifies discrete subpopulations of activated stromal cells across a wide range of tissue types and pathological contexts [22,23,24]. In human CAFs, a conserved TGFβ-driven LRRC15+ CAF population co-expresses myofibroblast markers including TAGLN and ACTA2 (in mouse), and is enriched for ECM-associated genes such as COL10A1, COL11A1, and MMP11 in human tumors [22]. Notably, this LRRC15-positive CAF signature is conserved across carcinomas, sarcomas, and other solid tumor types, pointing to a universal stromal activation program in response to tumor-derived cues [22].

In OS, scRNA-seq analyses have detected LRRC15 expression in both malignant tumor cells and stromal fibroblasts, and elevated expression is strongly correlated with a mesenchymal differentiation state and an invasive cellular phenotype [25,26]. In addition, LRRC15 expression in MSCs is regulated by the transcription factor TWIST1 [27], a master regulator of epithelial–mesenchymal transition (EMT) and stemness, linking LRRC15 to mesenchymal cell identity.

In osteoarthritis, RNA-seq analyses of human OA cartilage revealed LRRC15 enrichment in a chondrocyte subpopulation characterized by hypertrophic and ossification signatures, including expression of COL10A1, RUNX2, and MMP13 [28]. This establishes LRRC15 as a specific marker of pathological chondrocyte activation during the aberrant cartilage-to-bone transition that drives OA progression.

Beyond the skeletal system, LRRC15-positive myofibroblasts were identified in multiple fibrotic contexts, including cutaneous wound healing and pulmonary fibrosis, while cross-tissue fibroblast atlases have established LRRC15 as a conserved marker of TGFβ-driven myofibroblast differentiation across diverse organs [7,24,29].

### 2.4. Protein-Level Validation

While mRNA expression profiling yields critical insights into transcriptional regulation, protein-level validation is indispensable for establishing LRRC15 as a bona fide cell surface marker and viable therapeutic target.

In OS, IHC staining of tumor tissue microarrays (TMAs) has confirmed that LRRC15 protein expression occurs in the majority of cases, with cross-sample staining intensity ranging from weak to strong [16]. LRRC15 expression was observed in both tumor cells and stromal fibroblasts, with stromal expression correlating with a higher tumor grade and increased metastatic potential [16]. Flow cytometric analysis of dissociated OS xenografts further confirmed LRRC15’s localization to the cell surface, validating its accessibility for antibody-based therapeutic strategies [30].

In osteoarthritis, IHC staining of OA cartilage sections revealed that LRRC15 protein localized to chondrocytes in the deep zone and calcified cartilage layer—regions undergoing active ossification [28]. Co-staining with established hypertrophic markers RUNX2 and COL10A1 provided further confirmation that LRRC15-positive chondrocytes display a clear hypertrophic differentiation phenotype [31].

Across solid tumor types, IHC and immunofluorescence studies have consistently identified LRRC15 protein expression within the stromal compartment of breast, ovarian, pancreatic, and non-small cell lung cancers [6,32,33]. Single-cell RNA sequencing revealed that LRRC15+ CAFs co-express canonical myofibroblast markers, including ACTA2 (α-SMA) and TAGLN, consistent with a TGFβ-driven myofibroblastic activation state [22,23].

Critically, LRRC15 protein is largely absent from healthy epithelial tissues and hematopoietic cell populations, providing a highly favorable therapeutic window for LRRC15-targeted therapies with minimal risk of on-target, off-tumor toxicity.

## 3. The Molecular Structure and Structural Function of LRRC15

### 3.1. The Molecular Structure of LRRC15

LRRC15 adopts the canonical structural architecture of LRR-family proteins, which are repeat-protein scaffolds characterized by short (19–29 residue) sequence motifs arranged in tandem. These motifs fold into a characteristic right-handed horseshoe-shaped solenoid, with the concave surface formed by parallel β-strands serving as the primary protein–protein interaction interface, while the convex surface contains variable helical elements [1,34,35]. This versatile structural framework underpins diverse protein–protein interactions across the LRR superfamily, as exemplified by Toll-like receptors (TLR3, TLR4) that utilize their concave surfaces for specific ligand engagement [36,37].

**Figure 2:**
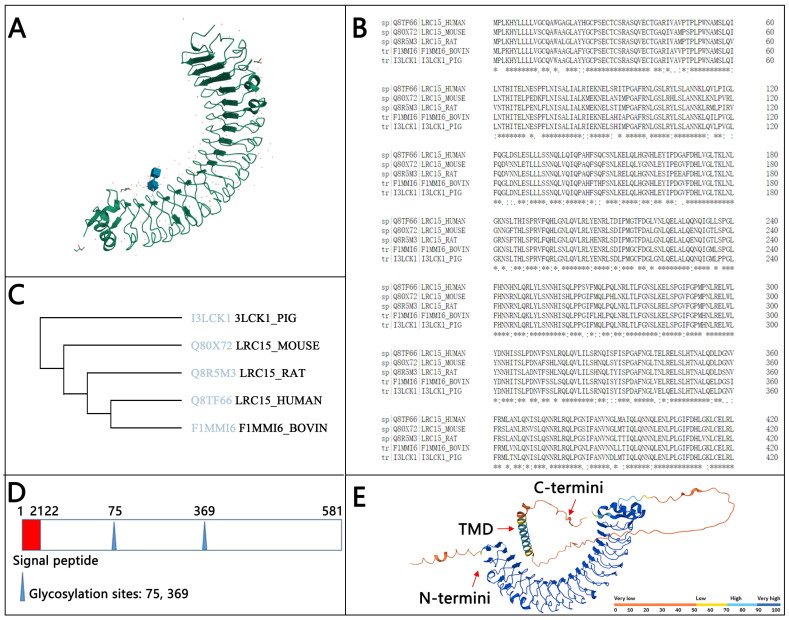
Sequence and structural analyses of LRRC15 protein. (**A**) Three-dimensional structure of mouse LRRC15 extracellular domain. (**B**) Multiple sequence alignment analyses show that LRRC15 shares amino acid sequence identity and similarity in humans, mice, rats, bovines, and pigs; these data were assembled at https://www.uniprot.org/align (asterisks, colons, and periods indicate fully conserved, strongly similar, and weakly similar residues, respectively, accessed on 10 March 2025). (**C**) A family tree of LRRC15 proteins. (**D**) Molecular structural analyses of LRRC15, predicted by bioinformatics information based on Uniprot (https://www.uniprot.org/(accessed on 9 June 2026)), showing that LRRC15 comprises a signal peptide and an LRRC15 protein with 2 putative glycosylation sites at amino acid residues 75 and 369, respectively. (**E**) Tertiary structure display of human LRRC15 predicted according to the AlphaFold Protein Structure Database (https://alphafold.ebi.ac.uk/entry/AF-Q8TF66-F1 (accessed on 9 June 2026)).

Until recently, experimental structural characterization of LRRC15 was hindered by challenges inherent to purifying type I transmembrane proteins with flexible membrane-proximal regions [6,38]. However, a recent study overcame these hurdles and assessed the experimental crystal structure of the mouse LRRC15 extracellular domain (PDB: 9OIB, https://www.rcsb.org/structure/9OIB (accessed on 9 June 2026)), resolved at 2.26 Å by X-ray crystallography (Figure 2A) [39]. Multiple sequence alignment analyses demonstrate that human LRRC15 shares approximately 75% sequence identity with orthologs from mouse, rat, bovine, and pig (Figure 2B,C), indicating high evolutionary conservation and validating the mouse structure as a reliable template for understanding the human protein [40]. This experimentally determined structure provides critical empirical validation for computational prediction models and delivers direct atomic-level insight into the molecular architecture of the LRRC15 extracellular domain.

Consistent with this cross-species conservation, the AlphaFold-predicted structure of human LRRC15 (https://alphafold.ebi.ac.uk/entry/AF-Q8TF66-F1 (accessed on 9 June 2026)) exhibits high structural congruence with the experimentally resolved mouse ectodomain. Human LRRC15 is a 581-amino acid type I transmembrane protein organized into six functional domains: (i) an extracellular N-terminal capping domain (residues 22–53); (ii) 15 leucine-rich repeats forming the horseshoe-shaped solenoid (residues 54–411), whose concave surface represents the predicted interaction interface for binding partners such as integrin β1; (iii) a C-terminal capping domain (residues 423–475); (iv) a flexible membrane-proximal stalk (residues 476–538); (v) a single transmembrane α-helix (residues 539–559); and (vi) a short cytoplasmic tail (residues 560–581) containing a palmitoylation-competent CCCC motif (Figure 2D,E) [6,40,41]. Notably, the protein lacks obvious intracellular signaling domains, suggesting that LRRC15 signals primarily through extracellular interactions with binding partners and lateral association with co-receptors such as integrins.

### 3.2. The Molecular Function of LRRC15

Like other LRR proteins, LRRC15 is involved in cell–cell and cell–extracellular matrix (ECM) interactions, including binding to transmembrane receptors or ligands, ECM constituents, and regulating inflammatory cytokine or growth factor-mediated pathways [5,13,42].

During fibroblastic differentiation, LRRC15 translocates to and accumulates at the plasma membrane. Its extracellular LRR domain directly engages multiple ECM constituents, particularly matrix collagens, and forms a physical complex with β1-integrin. This facilitates focal adhesion assembly and initiates downstream signaling through FAK, phosphatidylinositol 3-kinase (PI3K), and protein kinase B (AKT) [10]. The resulting LRRC15-FAK axis is a critical regulator of cell adhesion, directional migration, and anoikis resistance in diverse cell populations, and is indispensable in TGF-β1-driven fibroblastic differentiation of periodontal ligament cells [10,43]. TGF-β has been identified as the principal upstream regulator of LRRC15 expression. TGF-β signaling induces robust LRRC15 upregulation in both myofibroblasts and CAFs, where LRRC15 functions to sustain immunosuppressive stromal phenotypes and modulate the cytokine microenvironment [43,44]. Meanwhile, CAF-expressed LRRC15 enhances tumor cell invasiveness through activation of the Wnt/β-catenin pathway. Furthermore, elevated LRRC15 levels in fibroblast-like synoviocytes are associated with excessive pro-inflammatory cytokine production in rheumatoid arthritis [9,45].

## 4. LRRC15 in Skeletal Biology and Bone Metabolism

### 4.1. Osteogenic Differentiation and the Stemness of the Mesenchymal Stem Cells

Converging evidence from multiple independent studies establishes LRRC15 as a novel positive regulator of MSC osteogenic differentiation, acting through suppression of NF-κB signaling to promote osteoblast lineage commitment [12,27]. MSCs are multipotent somatic progenitor cells present in bone marrow, the umbilical cord, and adipose tissue [46], and the identification of LRRC15 as both an inducible marker and a functional mediator of their osteogenic differentiation represents a significant advance in understanding the molecular control of bone formation.

The core mechanistic evidence comes from loss- and gain-of-function studies demonstrating that LRRC15 is both necessary and sufficient for MSC osteogenic differentiation. LRRC15 mRNA and protein are progressively upregulated during osteogenic induction, paralleling canonical osteogenic markers including RUNX2, osterix (OSX), alkaline phosphatase (ALP), and osteocalcin (OCN) [12]. Critically, shRNA-mediated LRRC15 knockdown profoundly impairs osteogenic capacity—reducing ALP activity, mineralized nodule formation, and osteogenic gene expression—without affecting MSC proliferation or viability, indicating a specific role in lineage commitment rather than general cellular fitness [12]. In vivo subcutaneous implantation experiments confirmed that LRRC15 depletion significantly reduces bone-like tissue formation [12]. Importantly, LRRC15 depletion did not affect MSC proliferation or viability, which suggests that it plays a specific role in lineage commitment rather than general cellular fitness. In vivo subcutaneous implantation experiments confirmed that LRRC15 KD significantly reduced bone-like tissue formation, with decreased collagen deposition and OCN expression observed at the implantation site [12]. Mechanistically, LRRC15 promotes osteogenesis by functioning as a negative regulator of NF-κB signaling: LRRC15 retains the p65 subunit in the cytoplasm, thereby relieving NF-κB-mediated transcriptional repression of osteogenic genes such as RUNX2 [12].

Complementary findings link LRRC15 to mesenchymal stem cell identity and stemness. LRRC15-positive MSC populations isolated from human and mouse bone marrow exhibit high expression of stemness-associated transcription factors and therapeutic cytokines, suggesting that LRRC15 marks a functionally competent MSC subpopulation with enhanced regenerative potential [27]. The transcription factor TWIST1, a master regulator of mesenchymal cell identity, drives LRRC15 expression by binding directly to conserved E-box elements within the LRRC15 promoter [27]. Mechanistically, TWIST1 binds directly to conserved E-box elements within the LRRC15 promoter to drive transcriptional activation [27]. These findings provide additional biological context for the osteogenic role of LRRC15, as MSC stemness maintenance and lineage commitment are closely interlinked. Notably, while TGF-β signaling has been independently established as a potent inducer of LRRC15 expression in mesenchymal stromal cells and fibroblasts in the context of stromal activation and cancer-associated fibroblast (CAF) differentiation [6,23], further investigation is needed to determine whether the TGF-β–LRRC15 axis directly contributes to osteogenic commitment in bone marrow MSCs.

**Figure 3:**
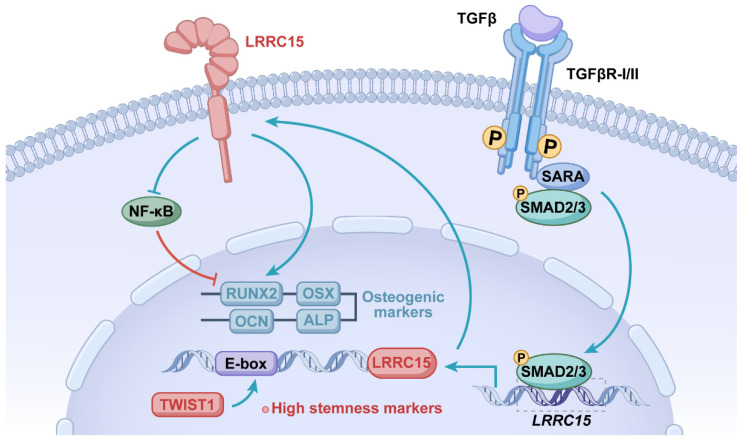
Molecular mechanisms of LRRC15 regulating the osteogenic differentiation of MSCs and its potential crosstalk with TWIST and TGF-β.

Collectively, these findings establish LRRC15 as a novel osteogenic regulator that promotes MSC osteoblast differentiation by suppressing NF-κB signaling. However, several key questions remain. It has yet to be fully determined whether LRRC15-positive MSCs—which exhibit high stemness marker expression [27]—represent the most functionally competent cell population for skeletal regenerative applications. Furthermore, the interplay between the TWIST1–LRRC15 stemness axis and osteogenic commitment pathways warrants additional investigation. Addressing these gaps will require direct comparative analyses of LRRC15 expression dynamics across MSC differentiation lineages and functional characterization of FACS-isolated LRRC15-positive versus negative MSC populations. To provide a visual illustration of these regulatory cascades and the underlying crosstalk, the predicted molecular mechanism is depicted in Figure 3.

### 4.2. Bone Formation and Remodeling

LRRC15 functions as a regulator of bone formation and remodeling in vivo, as demonstrated in the ovariectomized (OVX) mouse model of postmenopausal osteoporosis. In this well-established model, LRRC15 expression in bone marrow stromal cells is significantly downregulated relative to sham-operated controls, and this reduction correlates with key histomorphometric parameters of bone loss [12].

Importantly, restoration of LRRC15 expression can rescue pathological bone loss. Local transplantation of LRRC15-overexpressing MSCs into the femoral bone marrow cavity of OVX mice drove significant enhancement of bone formation relative to control MSCs, as confirmed by micro-CT and histomorphometric analyses. Histological evaluation demonstrated increased osteoblast numbers and osteoid surface area, consistent with enhanced osteoblast functional activity [12].

These results establish LRRC15 as a functional regulator of in vivo bone formation with potential therapeutic utility for enhancing bone regeneration in osteoporosis and fracture repair. However, these proof-of-concept experiments relied on local MSC transplantation rather than systemic modulation of LRRC15 activity, and the precise molecular mechanisms through which LRRC15 enhances osteoblast function in the in vivo bone microenvironment remain incompletely characterized.

LRRC15 expression is also dynamically regulated during fracture healing [47]. In a mouse tibial fracture model, LRRC15 mRNA and protein expression were markedly upregulated in the fracture callus during the early inflammatory and soft callus formation phases, with peak expression coinciding with maximal chondrogenesis and endochondral ossification [47]. Spatial transcriptomic and single-cell analyses of long bone fracture healing have further confirmed that LRRC15 is dynamically regulated across distinct healing stages, exhibiting particularly prominent expression in chondrogenic and osteogenic cell populations within the callus microenvironment [47]. Immunohistochemical staining localized LRRC15 protein to periosteal progenitor cells, hypertrophic chondrocytes within the cartilaginous callus, and osteoblasts at the ossification front, suggesting that LRRC15 plays a spatially coordinated role in directing the chondro-osteogenic transition during endochondral bone repair [47]. This spatiotemporal expression pattern suggests that LRRC15 participates in the coordinated cellular responses required for fracture repair, including mesenchymal cell recruitment, chondrogenesis, and osteogenesis.

### 4.3. Osteoporosis and Bone Loss

While the studies described above implicate LRRC15 as being involved in bone formation, direct evidence linking LRRC15 to osteoporosis pathogenesis in humans remains limited. Bioinformatic analyses have identified LRRC15 as differentially expressed in some cohorts, but the direction and magnitude of change vary across studies [31,48,49]. While some reports describe downregulation of LRRC15 in osteoporotic bone, consistent with impaired osteoblast activity, others have found no significant differences in expression between diseased and healthy tissue [48,49]. In OP models, LRRC15 expression is downregulated, aligning with its established role in promoting osteogenic differentiation and bone formation [48]. This expression pattern stands in direct contrast to the upregulation of LRRC15 observed in inflammatory joint diseases, highlighting the highly context-dependent nature of LRRC15 expression and function.

Genome-wide association studies of bone mineral density and osteoporotic fracture risk have not identified LRRC15 as a high-priority susceptibility locus, suggesting that common genetic variants within LRRC15 do not exert a strong effect on population-level fracture risk [50]. However, rare variants or epigenetic modifications that affect LRRC15 expression may contribute to individual susceptibility, warranting further investigation.

From a mechanistic perspective, the role of LRRC15 in osteoporosis is likely linked to its core functions in regulating osteoblast differentiation and functional activity. Reduced LRRC15 expression in bone marrow stromal cells—driven by factors such as aging, estrogen deficiency, and chronic inflammatory cytokine exposure—may impair osteoprogenitor commitment and de novo bone formation, contributing to the pathological imbalance between bone resorption and formation that defines osteoporosis [51]. Therefore, therapeutic strategies designed to restore LRRC15 expression or function in the osteoblast lineage hold theoretical potential to enhance bone formation and reduce fracture risk, though such approaches have yet to progress beyond the preclinical proof-of-concept stage.

## 5. LRRC15 in Joint Diseases

### 5.1. LRRC15 in Osteoarthritis

LRRC15 has emerged as a key molecular driver of pathological cartilage ossification and ECM remodeling, and infrapatellar fat pad fibrosis in OA [13,28,31]. OA is a degenerative joint disease characterized by progressive cartilage degradation, subchondral bone remodeling, synovial inflammation, and pain [52]. Recent studies have identified LRRC15 as a key player in OA pathogenesis, particularly in the process of pathological cartilage ossification and extracellular matrix remodeling [28,31].

Bulk gene expression profiling of human OA cartilage relative to healthy articular cartilage has demonstrated significant upregulation of LRRC15 mRNA in OA tissue. scRNA-seq profiling of dissociated human OA cartilage further identified marked enrichment of LRRC15 in a specific chondrocyte subpopulation, defined by the expression of hypertrophic differentiation markers (including COL10A1, RUNX2, MMP13, and IHH) and pro-ossification genes (such as SPP1, IBSP, and ALPL) [28]. This LRRC15-positive chondrocyte population is predominantly localized to the deep zone and calcified layer of OA cartilage, which are the regions of the tissue that undergo active endochondral-like ossification during disease progression.

IHC staining of human OA cartilage sections confirmed the presence of LRRC15 protein expression in chondrocytes within the deep cartilage zone and at the cartilage–bone interface [13]. Transcriptomic analysis and functional experiments identified LRRC15 as a positive regulator of chondrocyte hypertrophic differentiation, with LRRC15 expression positively correlating with the canonical hypertrophic markers RUNX2 and COL10A1; notably, LRRC15 silencing significantly suppressed expression of these hypertrophic markers [53]. In addition, correlative analyses have shown that LRRC15 protein expression levels correlate positively with OA histological severity, with markedly higher expression observed in advanced-stage disease relative to early-stage OA [31].

Functionally, LRRC15 acts as a key driver of chondrocyte activation and aberrant ECM remodeling in OA. LRRC15 overexpression in chondrocytes enhanced the expression of matrix metalloproteinases (MMP1, MMP3, MMP13) and aggrecanases (ADAMTS4, ADAMTS5), enzymes which are responsible for cartilage ECM degradation [13]. Conversely, LRRC15 knockdown in chondrocytes reduced MMP expression and attenuated IL-1β-induced cartilage degradation in ex vivo cartilage explant cultures [53].

Targeting LRRC15 in OA represents a potential therapeutic strategy. Based on the established role of LRRC15 in promoting catabolic signaling in OA chondrocytes, pharmacological or antibody-mediated blockade of LRRC15 may attenuate downstream integrin–FAK–NF-κB signaling and reduce cartilage degradation, although specific in vivo proof-of-concept studies in standardized OA models remain to be conducted.

### 5.2. LRRC15 in Rheumatoid Arthritis

Recent studies have identified LRRC15 as both a marker and a functional regulator of activated FLSs in RA [14]. RA is a chronic systemic autoimmune disease defined by progressive synovial inflammation, irreversible cartilage and bone destruction, and extra-articular systemic complications [54]. FLSs are well-established key effector cells in RA pathogenesis which are responsible for the production of pro-inflammatory cytokines, matrix-degrading enzymes, and the direct mediation of joint tissue destruction [55].

Transcriptomic profiling of synovial tissue from patients with RA, relative to tissue from OA patients and healthy controls, has revealed significant upregulation of LRRC15 mRNA specifically in RA synovium [14]. Single-cell RNA-seq analysis of RA synovium identified LRRC15 expression in a subset of FLSs characterized by high expression of inflammatory cytokines (IL6, IL8, CXCL12), matrix metalloproteinases (MMP1, MMP3), and fibroblast activation markers (PDPN, THY1, FAP) [14]. This LRRC15+ FLS population was expanded in RA compared to OA and healthy controls, suggesting that it has a pathogenic role [14].

IHC staining of RA synovial tissue confirmed LRRC15 protein expression in the synovial lining layer and sublining stromal compartment, with co-localization to vimentin+ and α-SMA+ fibroblasts [14]. Flow cytometry analysis of dissociated RA synovial cells demonstrated that LRRC15+ FLSs represent 15–30% of total FLSs, with higher proportions in patients with active disease compared to those in remission [14].

RUNX1 has been identified as a key transcriptional and epigenetic regulator of LRRC15 expression in RA fibroblast-like synoviocytes (FLSs) [14]. RUNX1 (Runt-related transcription factor 1) is a master regulator of hematopoietic and mesenchymal cell differentiation, and its dysregulation has been implicated in RA pathogenesis [56]. RUNX1 binds directly to the LRRC15 promoter at multiple consensus RUNX binding sites (5′-TGTGGT-3′), activating transcription in RA FLSs [14]. Chromatin immunoprecipitation sequencing provided confirmation of RUNX1 occupancy at the LRRC15 locus, with enrichment of active histone marks (H3K4me3, H3K27ac) in RA FLSs compared to healthy FLSs [14].

Functionally, RUNX1-mediated LRRC15 upregulation promotes FLS activation and inflammatory cytokine production. RUNX1 knockdown in RA FLS reduced LRRC15 expression by ~70% and also led to a significant decrease in IL-6, IL-8, and MMP3 secretion [14]. Conversely, RUNX1 overexpression in healthy FLSs induced LRRC15 expression and conferred an RA-like inflammatory phenotype [14]. These findings have established a RUNX1-LRRC15 regulatory axis as a key driver of FLS activation in RA.

In vivo studies using the well-established collagen-induced arthritis (CIA) mouse model have demonstrated that pharmacological inhibition of RUNX1 (using the small-molecule inhibitor Ro5-3335) reduces LRRC15 expression in synovial tissue, markedly attenuates joint inflammation, and ameliorates cartilage and bone destruction [14]. Histological analysis revealed reduced synovial hyperplasia, decreased inflammatory cell infiltration, and lower OARSI scores in Ro5-3335-treated mice compared to vehicle controls [14]. This epigenetic repression thus reduces LRRC15 levels and inhibits RA development. The effects of RUNX1 on RA pathology are reversed by LRRC15 overexpression or inhibition of the RUNX1–CBF-β complex, confirming that LRRC15 is a critical downstream target of RUNX1 in RA. In support of a potential reciprocal relationship, enforcing expression of *lrrc15* in wild-type zebrafish embryos reduced *runx1* expression in the dorsal aorta and *gata1a* in hematopoietic domains [57]. These findings suggest that targeting the RUNX1-LRRC15 axis may represent a novel therapeutic strategy for RA.

## 6. LRRC15 in Osteosarcoma Biology and Tumor Microenvironment

### 6.1. Expression Patterns and Clinical Implications

OS is the most common primary malignant bone tumor in children and adolescents, characterized by high metastatic potential and poor survival rates despite aggressive multimodal therapy [58]. LRRC15 has emerged as a critical biomarker and therapeutic target in OS, with expression in both tumor cells and stromal compartments [15,16].

Analysis of The Cancer Genome Atlas (TCGA) and TARGET OS datasets revealed significantly elevated LRRC15 mRNA in OS tissue relative to normal bone, and IHC staining of tissue microarrays confirmed protein-level expression in the majority of cases with variable staining intensity [16]. Notably, stromal LRRC15 expression specifically correlates with higher tumor grade and enhanced metastatic potential; tumors with high stromal LRRC15 exhibit significantly higher rates of pulmonary metastasis at diagnosis and shorter metastasis-free survival [16].

Importantly, LRRC15 protein was detected in both malignant tumor cells and intratumoral stromal fibroblasts, with stromal LRRC15 expression specifically correlating with a higher tumor grade and enhanced metastatic potential [16]. Tumors with high stromal LRRC15 expression exhibit significantly higher rates of pulmonary metastasis at diagnosis and shorter metastasis-free survival relative to tumors with low stromal LRRC15 expression.

scRNA-seq analyses of primary OS tumors have provided additional evidence of heterogeneous LRRC15 expression across distinct CAFs [23]. Single-cell transcriptomic analysis of pediatric OS tumors identified recurrent lineage-associated tumor cell states—comprising osteoblastic-like, chondroblastic-like, and fibroblastic-like programs—with LRRC15 exhibiting variable expression across these subpopulations as a candidate surface immunotherapeutic target [59]. Notably, functional perturbation studies indicate that LRRC15 may support osteogenic transcriptional programs in OS cells, as LRRC15 knockdown was associated with reduced expression of the osteoblastic transcription factors RUNX2 and SP7 [60]. Thus, LRRC15 was established as a marker of a dedifferentiated, highly invasive OS cell state that is strongly associated with poor clinical outcomes.

### 6.2. Functional Roles in Invasion, Metastasis, and Differentiation

In OS cells, LRRC15 functions as a key cell adhesion mediator that is essential for maintaining intercellular communication, cellular invasion, and differentiation [61,62], while its depletion disrupts ECM integration, leading to reduced adhesion to ECM components and impaired cell migration. In in vivo models, inhibiting LRRC15 reduces metastatic dissemination, indicating that it promotes cell adhesion and invasion through ECM interactions [10]. Across various cancers, high LRRC15 expression correlates with poor immunotherapy response, enhanced tumor progression, and increased metastasis [10], while human genetic studies have linked LRRC15 variants to elevated metastatic risk, suboptimal chemotherapeutic response, and poor survival, supporting its functional relevance in skeletal biology [16].

Functional studies using inducible LRRC15 knockdown (KD) model systems have provided evidence that LRRC15 is a critical regulator of OS cell adhesion, invasion, and differentiation [60]. Under 2D culture conditions (in which cells were grown in a flat monolayer), LRRC15 KD OS cells exhibited significantly reduced proliferation, increased apoptosis, and impaired migration compared to control cells, and in Transwell invasion assays, LRRC15 KD cells exhibited markedly reduced invasive capacity, indicating that LRRC15 is essential for OS cell invasion [16]. In 3D culture systems (involving cells grown in 3D spheroids), including spheroid and patient-derived organoid models, LRRC15 KD OS cells form smaller, less cohesive tumor spheroids, with significantly reduced overall viability and increased apoptosis within the spheroid core [60]. Live-cell imaging of these 3D cultures revealed that LRRC15 KD spheroids display profound defects in cell–cell adhesion, with increased spontaneous cell detachment and a reduced intercellular contact area [60]. These findings firmly establish LRRC15 as a critical cell adhesion molecule that is required for maintaining intercellular communication within the 3D OS tumor microenvironment.

At the molecular level, LRRC15 drives OS cell invasion via the integrin–FAK-SRC signaling axis. LRRC15 KD in OS cells results in reduced FAK phosphorylation, impaired SRC activation, and diminished downstream signaling through the ERK1/2 and AKT pathways. Rescue experiments demonstrated that re-expression of wild-type LRRC15 fully restores FAK phosphorylation and invasive capacity, whereas re-expression of an LRRC15 mutant lacking the extracellular LRR domain fails to rescue these phenotypes [63]. These data confirm that the extracellular LRR domain of LRRC15 is critical to its pro-invasive function.

In vivo studies using orthotopic and subcutaneous OS xenograft models confirmed LRRC15’s role in tumor growth and metastasis. LRRC15 KD 143B cells injected into the tibiae of NOD/SCID mice formed significantly smaller primary tumors and exhibited markedly reduced lung metastasis compared to control cells [15]. Histological analysis revealed that LRRC15 KD tumors exhibited reduced proliferation (Ki67 staining), increased apoptosis (TUNEL staining), and decreased microvessel density (CD31 staining) indicative of impaired tumor angiogenesis [64].

### 6.3. Prognostic Value and Biomarker Potential

Multiple independent clinical cohorts consistently demonstrate that LRRC15 is a robust, independent prognostic biomarker in OS. High LRRC15 mRNA expression is associated with significantly shorter overall survival in the TARGET OS cohort, and this association remains significant after multivariate adjustment for age, sex, tumor size, and metastatic status [16]. Concordant results from a OS cohort confirmed that high LRRC15 protein expression predicts both shorter overall survival and shorter metastasis-free survival [16].

The prognostic value of LRRC15 is particularly pronounced in patients with localized disease at diagnosis, suggesting utility for identifying patients at high risk of occult metastasis who may benefit most from intensified adjuvant therapy [16]. Additionally, LRRC15 expression correlates with chemotherapy response: patients with high expression exhibit poorer histological response to neoadjuvant chemotherapy and higher rates of chemoresistance, potentially via integrin-mediated/cell adhesion-mediated drug resistance (CAM-DR) mechanisms [16].

Collectively, these findings support the clinical utility of LRRC15 expression profiling for patient risk stratification and treatment personalization. LRRC15 could be incorporated into existing prognostic nomograms or used to identify patients most likely to benefit from LRRC15-targeted therapeutic strategies.

## 7. LRRC15 and Signaling Pathways in the Skeletal System

### 7.1. NF-κB Signaling

Evidence from multiple studies indicates that LRRC15 functions as a context-dependent, bidirectional regulator of NF-κB signaling in the skeletal system, exerting inhibitory or stimulatory effects that diverge sharply across MSCs, tumor-associated stroma, and inflammatory synovial fibroblasts [12,45,63]. The nuclear factor-κB (NF-κB) signaling pathway serves as a master regulator of inflammation and tissue catabolism in the musculoskeletal system. In skeletal diseases, including OA, RA, intervertebral disk degeneration, and OS, the canonical NF-κB pathway is potently activated by upstream stimuli such as pro-inflammatory cytokines (TNF-α, IL-1β) and damage-associated molecular patterns (DAMPs) [62,65]. Upon activation, IκBα is phosphorylated by the IκB kinase (IKK) complex and subsequently degraded by the proteasome, liberating the p65/p50 heterodimer to translocate into the nucleus, where it drives the transcription of downstream target genes encoding pro-inflammatory cytokines (e.g., IL-6, IL-8), matrix metalloproteinases, and other mediators of ECM catabolism—collectively perpetuating a self-amplifying cycle of tissue destruction and cellular dysfunction [65].

The connection between LRRC15 and NF-κB signaling was initially suggested by neurological studies identifying LRRC15 as a β-amyloid-inducible gene in astrocytes [5], with subsequent work demonstrating NF-κB-mediated modulation of amyloid pathology [66]. While these observations were made in a non-skeletal context, they provided the initial conceptual framework for investigating LRRC15–NF-κB crosstalk—a relationship that has since been substantiated in multiple skeletal cell types with important functional consequences.

In the context of MSC osteogenic differentiation, the LRRC15 functions as a negative regulator of NF-κB signaling in the skeletal system [12]. Lentiviral shRNA-mediated LRRC15 knockdown in human BMSCs promoted cytoplasmic-to-nuclear translocation of the p65 subunit, enhancing NF-κB transcriptional activity and profoundly impairing osteogenic differentiation; conversely, LRRC15 overexpression allowed p65 to be retained in the cytoplasm, relieving NF-κB-mediated transcriptional repression of osteogenic genes and facilitating osteoblast differentiation [12].

In contrast to the suppressive role of LRRC15 in MSCs, LRRC15 exerts pro-inflammatory and pro-tumorigenic effects by promoting NF-κB activation in pathological settings characterized by chronic inflammation and dysregulated cell proliferation, including rheumatoid arthritis and osteosarcoma [45,63]. This functional dichotomy reflects the cell type- and context-dependent nature of LRRC15 signaling, with distinct downstream consequences in inflammatory synovial fibroblasts versus cancer-associated stromal cells.

In RA synovial fibroblasts (FLSs), LRRC15 exerts a pro-inflammatory function by promoting NF-κB pathway activation. Recent research demonstrated that in RA FLSs, IL-1β stimulation upregulated LRRC15 expression alongside elevated protein levels of NF-κB p65, phosphorylated p65 (p-p65), and phosphorylated IκBα (p-IκBα), with concomitant downregulation of total IκBα and Nrf2 [45]. Critically, LRRC15 silencing in RA FLSs reversed these molecular changes, reducing IL-1β-induced NF-κB activation and attenuating the downstream production of pro-inflammatory mediators such as IL-6, IL-8, and MMP3 [45].

Within the tumor-associated fibroblast compartment of OS, LRRC15 assumes a distinctly pro-tumorigenic function as a surface marker enriched in myofibroblastic CAFs, emerging as a critical regulator of the immunosuppressive tumor microenvironment [63]. Mechanistically, LRRC15 exerts its pro-tumorigenic effects through activation of the FAK/SRC/NF-κB signaling axis in CAFs [63]. Upon LRRC15 engagement, downstream integrin signaling is triggered, leading to sequential phosphorylation of FAK and proto-oncogene tyrosine–protein kinase SRC [63]. This phosphorylation cascade ultimately converges on the activation of NF-κB, specifically via phosphorylation of the p65 subunit [63]. Activated NF-κB translocates to the nucleus and transcriptionally upregulates interleukin-8 (IL-8) expression [63,67]. The secreted IL8 then acts in a paracrine manner to promote macrophage chemotaxis and drive their polarization toward the immunosuppressive M2-like phenotype, characterized by elevated CD206 expression and enhanced TGF-β secretion [63,68]. Pharmacological inhibition of FAK (PF573228), SRC (PP2 or dasatinib), or NF-κB (CAPE) effectively abrogates LRRC15-induced IL8 upregulation and subsequent M2 macrophage polarization, confirming the functional significance of this LRRC15-NF-κB-IL8 signaling cascade [63]. Notably, this pathway forms part of a positive feedback loop wherein M2-like macrophages, in turn, secrete TGF-β1 that further induces LRRC15 expression in CAFs via SMAD2-dependent transcriptional activation.

**Figure 4:**
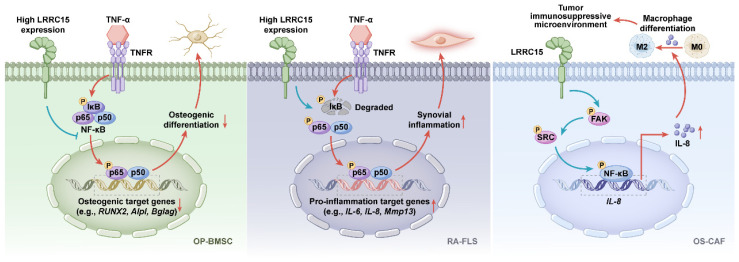
Schematic molecular mechanism of LRRC15-mediated regulation of the NF-κB signaling pathway in skeletal system.

Collectively, the evidence above reveals that LRRC15 exerts context-dependent, bidirectional effects on NF-κB signaling (Figure 4). In BMSCs, LRRC15 functions as a negative regulator of NF-κB by retaining p65 in the cytoplasm, thereby promoting osteogenic commitment and bone formation. In cancer-associated fibroblasts (CAFs), LRRC15 drives NF-κB activation through the FAK/SRC signaling axis, leading to transcriptional upregulation of IL-8 and subsequent M2-like macrophage polarization that fosters an immunosuppressive tumor microenvironment. Similarly, in RA FLSs, LRRC15 promotes NF-κB activation and downstream production of inflammatory cytokines and matrix-degrading enzymes, perpetuating synovial inflammation. This cell type- and context-specific duality likely reflects differences in the molecular interactome of LRRC15, the prevailing inflammatory microenvironment, and the availability of co-regulatory transcription factors in each cellular context. These findings underscore the complexity of therapeutically targeting the LRRC15–NF-κB axis and highlight the need for cell type-specific strategies to harness the beneficial effects of LRRC15 modulation across different pathologies.

### 7.2. TGF-β Signaling

LRRC15 has been identified as a direct transcriptional target of canonical TGF-β signaling, and functions as a key downstream effector of TGF-β-induced fibroblast-to-myofibroblast transition [6,19,23]. TGF-β is a master regulator of fibroblast activation, extracellular matrix production, and tissue fibrosis [69]. Mechanistically, the extracellular domain of LRRC15 directly engages TGF-β receptor II to stabilize the TGF-β ligand–receptor complex, leading to a substantial enhancement in the phosphorylation efficiency of downstream Smad2/3 [70]. Multiple studies have demonstrated that TGF-β1 treatment induces LRRC15 expression in diverse cell types, including mesenchymal stem cells, fibroblasts, and cancer-associated fibroblasts [6]. In human bone-marrow-derived MSCs, TGF-β1 induces LRRC15 mRNA expression in a dose- and time-dependent manner [12].

Consistent with these findings, multiple studies have confirmed that LRRC15 is transcriptionally upregulated in both myofibroblasts and CAFs in response to TGF-β stimulation. Dexamethasone—a known activator of the TGF-β pathway—similarly elevates LRRC15 levels in osteoblasts, while TGF-β treatment of human lung fibroblasts and corneal fibroblasts induces robust LRRC15 upregulation [6,12]. At the level of cell fate determination, TGFβR2 signaling in dermatopontin-positive universal fibroblasts drives differentiation into LRRC15+ myofibroblasts, establishing LRRC15 as a key marker of fibroblast-to-myofibroblast transition [23]. In the cancer context, TGF-β drives the LRRC15+ CAF phenotype in pancreatic cancer, where these fibroblasts promote tumor progression and immunotherapy resistance [22]. Collectively, these findings position LRRC15 as a critical mediator of TGF-β’s pro-fibrotic and stromal activation effects across both physiological and pathological contexts.

### 7.3. Integrin β1 and Focal Adhesion Kinase (FAK) Signaling

LRRC15 emerges as a critical regulator of cell–ECM adhesion and mechanotransduction in the skeletal system through direct binding to integrin β1 and subsequent activation of the FAK signaling cascade, thereby governing cell adhesion, migration, and invasive behavior across both stromal and malignant contexts [10,19]. It is well-established that integrin signaling pathways serve as a critical mechanical and chemical link between the extracellular matrix and the actin cytoskeleton, transducing extracellular cues into intracellular signals that drive a wide range of downstream cellular functions [71,72]. Integrin β1 (ITGB1) is the most ubiquitously expressed integrin β subunit, forming functional heterodimers with multiple α subunits (α1-α11, αV) to recognize a diverse array of ECM ligands, including collagen, fibronectin, and laminin [27,73,74].

Through its extracellular leucine-rich repeat domains, LRRC15 directly interacts with integrins to synergistically engage core ECM components, including fibronectin, laminin, and collagen, and to modulate bidirectional cell–ECM communication [10,19]. Notably, β1-integrin is one of the most well-studied integrins that interacts with LRRC15, and it can heterodimerize with several α-subunits to form receptors for diverse ligands [27,73,74]. LRRC15 has been reported to regulate the expression of multiple ECM-related genes by directly binding to and activating β1-integrin [10].

During this process, LRRC15-expressing cells adhere to fibronectin and promote integrin β1 clustering at the cell surface, driving activation of the FAK signaling pathway and the formation of mature focal adhesions (FAs) [10]. FAs are stable, multi-protein ECM adhesion structures whose formation enhances the migratory and invasive capacity of tumor cells, promoting metastatic dissemination. As integrin engages with ECM ligands, FAK undergoes autophosphorylation at tyrosine 397, creating a high-affinity binding site for SRC family kinases, which subsequently phosphorylate FAK at additional tyrosine residues to drive its full activation [75]. The fully activated FAK then phosphorylates a range of downstream signaling targets, including paxillin, p130Cas, and ERK1/2, to promote cell adhesion, migration, and survival [75].

The functional importance of the LRRC15–integrin–FAK axis has been demonstrated across multiple cell types. In periodontal ligament fibroblasts, LRRC15 is essential for TGF-β1-induced FAK phosphorylation; its depletion significantly attenuates FAK activation and downstream SRC/ERK1/2 signaling [19]. In TNBC, LRRC15 promotes proliferation and invasion through the ITGB1/FAK/PI3K pathway, with LRRC15-overexpressing cells exhibiting enhanced adhesive phenotypes that contribute to β1-integrin-mediated metastasis [10,74]. In addition, the LRRC15-overexpressing OVCAR7 cell line exhibited an enhanced adhesive phenotype which contributed to β1-integrin-mediated ovarian cancer metastasis, whereas LRRC15-knockdown cells showed reduced adhesive properties [10]. Moreover, in both CAFs and tumor cells, LRRC15 binds β1-integrin and enhances its activation and clustering at the cell surface [10,74].

Mechanistically, LRRC15 may promote FAK activation by stabilizing integrin β1 clustering at focal adhesions, thereby enhancing integrin-mediated “outside-in” signaling. Alternatively, LRRC15 may directly recruit FAK or adaptor proteins to focal adhesions through its intracellular domain. Further structural and biochemical studies are needed to elucidate the precise molecular mechanisms.

In OS, the LRRC15-integrin-FAK signaling axis is a key driver of tumor cell invasion and metastatic dissemination [27,60]. LRRC15 knockdown in OS cells reduced FAK phosphorylation, impaired cell adhesion to ECM substrates (collagen I, fibronectin), and decreased invasive capacity [27,60]. Pharmacological inhibition of FAK phenocopied LRRC15 knockdown, reducing OS cell invasion and metastasis in vitro and in vivo. These findings firmly establish the LRRC15-FAK signaling axis as a highly promising druggable pathway in OS.

Collectively, the evidence delineates a conserved LRRC15–integrin β1–FAK mechanotransduction axis that reinforces cell–ECM adhesion and drives migratory and invasive phenotypes across both stromal and malignant cellular contexts, rendering it an attractive therapeutic target for curtailing metastatic progression, particularly in OS.

### 7.4. Wnt/β-Catenin Signaling

LRRC15 has emerged as a putative regulator of canonical Wnt/β-catenin signaling primarily from oncological studies, where it promotes β-catenin nuclear translocation to drive tumor progression, whereas its functional involvement in skeletal Wnt signaling and bone homeostasis remains a critical unresolved gap [9]. The canonical Wnt signaling pathway is activated by Wnt protein ligands binding to Frizzled receptors and LRP5/6 co-receptors on the cell surface, which drives the phosphorylation of β-catenin and activates downstream signaling—particularly the upregulation of osteogenesis- or mineralization-related genes [76]. It directly modulates the differentiation of MSCs into osteoblasts and indirectly participates in bone resorption by crosstalking with the OPG/RANK/RANKL pathway [77].

Previous studies have reported that Wnt signaling activation is involved in TNBC metastasis [78], as it induces epithelial-to-mesenchymal transition and cisplatin resistance [79]. Consistently, LRRC15 is highly expressed in TNBC tissues and is associated with poor prognosis [9]. Furthermore, bioinformatic analyses have revealed a positive correlation between LRRC15 and COL10A1 expression [80]. As well as being related to the calcification of breast cancer [81], COL10A1 is also a significant biomarker for chondrocyte hypertrophy and cartilage calcification [82], and it can be upregulated by the canonical Wnt pathway [83]. This indicates that a potential regulatory network may exist between LRRC15, the Wnt/β-catenin pathway, and COL10A1 that can modulate cell hypertrophy or calcification. In the tumor microenvironment, Yang et al. discovered that overexpressed LRRC15 promotes β-catenin nuclear translocation by disrupting its binding to Axin1, thereby inducing tumor metastasis [9]. However, whether LRRC15 modulates β-catenin in skeletal biology has not been elucidated.

Notably, direct experimental evidence linking LRRC15 to the regulation of Wnt signaling in osteoblasts is currently lacking, representing a critical and unresolved gap in our understanding of LRRC15’s function in the skeletal system. This topic should be prioritized in future investigations.

## 8. LRRC15 as a Potential Therapeutic Target for the Treatment of Skeletal Diseases

LRRC15 serves as both a validated biomarker and a functionally active driver across multiple skeletal pathologies, positioning it as a compelling cross-disease therapeutic target. Beyond oncology, LRRC15 is functionally implicated in bone metabolism; its knockdown impairs osteogenic differentiation of bone marrow-derived MSCs, contributing to conditions such as postmenopausal osteoporosis [12]. In a mouse model of OA, LRRC15 downregulation significantly reduces the expression of genes (e.g., MMP10, Nos2, and Ptgs2) involved in cartilage catabolism induced by chronic IL-1β stimulation, as well as the expression of genes associated with a myofibroblast phenotype and ECM-producing signature, including ACTA2, FN, and COL1A1 [13]. Similarly, in rheumatoid arthritis (RA), LRRC15 inhibition slows disease progression [14,28].

The development of antibody–drug conjugates (ADCs) that bind LRRC15 to deliver cytotoxic agents specifically to tumor cells or the tumor microenvironment (TME) represents a promising therapeutic strategy for skeletal malignancies [10,84]. ABBV-085, a new type of ADC, is a humanized anti-LRRC15 antibody comprising hydrophobic interaction chromatography-enriched E2, with two anti-mitotic monomethyl auristatin E (MMAE) payloads conjugated per antibody [6]. Upon binding to LRRC15-positive stroma, cell-permeable MMAE diffuses into adjacent tumor cells, eliciting a potent bystander effect that eliminates proliferating cancer cells and suppresses tumor growth. ABBV-085 has demonstrated efficacy in xenograft models of various solid tumors, including sarcoma, breast, lung, pancreatic, and ovarian cancers [10,15,85]. Notably, while ABBV-085 strongly inhibits rapidly proliferating stromal cells in tumors, it shows minimal toxicity toward normal, slowly dividing LRRC15-positive MSCs or fibroblasts, mitigating concerns about on-target toxicity in healthy tissues [6]. LRRC15-positive MSCs, which have a low proliferative rate, exhibited minimal sensitivity to ABBV-085 in vitro, alleviating concerns regarding potential normal tissue toxicity from targeting these MSCs. In OS patient-derived xenograft (PDX) studies by the Pediatric Preclinical Testing Consortium, ABBV-085 inhibited tumor growth in PDX models and significantly improved event-free survival [15]. A phase I clinical trial further confirmed its safety and preliminary antitumor activity in patients with LRRC15-expressing OS and undifferentiated pleomorphic sarcoma [84]. Thus, as a novel marker for TGF-β-activated fibroblasts and mesenchymal stem cells, LRRC15 may serve as a therapeutic target via ABBV-085 for LRRC15-positive stromal desmoplasia or cancers of mesenchymal origin [6]. However, there is insufficient evidence to demonstrate the effectiveness of ABBV-085 for non-tumor skeletal system diseases. Given the limited expression of LRRC15 in normal tissues and its significant upregulation in certain skeletal diseases, targeting LRRC15 in the treatment of skeletal diseases has emerged as a potential research direction, as researchers have begun to focus on developing novel ADCs by coupling cartilage-protective drugs for OA or bone-anabolic drugs for osteoporosis.

In recent years, natural compounds derived from plants and other natural sources have attracted increasing attention for their potential as treatments for skeletal disease. Brevilin A (BrA), a sesquiterpene lactone isolated from Centipeda minima and identified as a novel LRRC15 inhibitor, exerts potent anti-RA effects by inhibiting the LRRC15/STAT3 signaling pathway [86]. Specifically, BrA’s capacity to covalently bind IKKα/β via its electrophilic α,β-unsaturated ketone moiety to suppress NF-κB signaling—an essential pathway in cartilage catabolism and synovial inflammation—complements its inhibition of the LRRC15/STAT3 axis, enabling synergistic suppression of multiple pathogenic processes in OA and RA [86,87]. Furthermore, BrA’s natural origin confers inherent biocompatibility [86], which is a critical advantage for long-term management of chronic skeletal disorders, while its well-documented ability to inhibit abnormal cell proliferation and induce apoptosis (e.g., in RA fibroblast-like synoviocytes and OS cells) extends its relevance to LRRC15-associated conditions beyond inflammatory arthritis, such as OS, in which LRRC15 serves as a validated biomarker. These findings provide opportunities for medicinal chemistry efforts to develop optimized LRRC15 inhibitors with improved potency, selectivity, and pharmacokinetic properties. Small-molecule inhibitors may offer advantages over antibody-based therapies, including oral bioavailability and lower cost.

Though significant progress has been made, the full scope of LRRC15 biology remains to be elucidated. Its evolutionary conservation and dynamic regulation suggest that it plays a fundamental role in critical biosynthetic and signaling networks [5]. In the skeletal system, LRRC15 appears to bridge protein-mediated inflammatory responses with complex cellular processes; however, the precise mechanisms by which it engages integrin, NF-κB, and Wnt pathways in skeletal pathophysiology require further clarification. Deeper investigation into LRRC15 expression patterns and its downstream signaling cascades will provide novel insights into the mechanisms of orthopedic diseases. This knowledge may accelerate the development of LRRC15-targeted diagnostic and prognostic tools, as well as innovative therapeutics for a range of skeletal disorders.

## 9. Summary

LRRC15, a transmembrane protein belonging to the LRR superfamily, mediates cell–cell and cell–extracellular matrix interactions. It participates in fibrogenesis, antiviral responses, and tumorigenesis by regulating signaling pathways such as NF-κB and Wnt/β-catenin. Enriched in musculoskeletal tissues and OS cells, LRRC15 enhances osteogenic differentiation of mesenchymal stem cells to alleviate osteoporosis, drives IL-1β-induced chondrocyte catabolism in OA, and promotes synovial inflammation in rheumatoid arthritis. As a prognostic biomarker for OS, it facilitates tumor invasion. The anti-LRRC15 antibody–drug conjugate ABBV-085 and BrA shows therapeutic potential in skeletal disease. Future studies clarifying its precise mechanisms may accelerate the development of targeted therapies for both tumor- and non-tumor-related skeletal diseases.

## Data Availability

No new data were created or analyzed in this study. Data sharing is not applicable to this article.
